# Poor efficacy of intravenous thrombolysis in de Winter pattern: A case report

**DOI:** 10.1097/MD.0000000000036270

**Published:** 2023-12-01

**Authors:** Hao Xiao, Zhao Mei, Zhang Feifei, Liu Huiliang, Li Shuren

**Affiliations:** a Hebei General Hospital, Shijiazhuang City, Hebei Province, China.

**Keywords:** de Winter pattern, intravenous thrombolysis, primary percutaneous intervention

## Abstract

**Introduction:**

Majority of patients with acute coronary syndrome can be quickly identified by electrocardiogram, but there are still 30% of patients with acute coronary artery lesions that cannot be recognized by electrocardiogram in time, resulting in delayed treatment.

**Patient concerns:**

Due to its special manifestations, de Winter syndrome is easily ignored by clinicians.

**Diagnosis:**

In this article we report a case of de Winter syndrome with poor thrombolytic effect to explore the optimal emergency management strategy for this patient.

**Interventions:**

The patient underwent remedial percutaneous coronary intervention (PCI) immediately after diagnosis.

**Outcomes:**

Patients recover well after PCI.

**Conclusion:**

de Winter syndrome is a strong indication of severe coronary artery disease, requiring rapid identification and opening of coronary vessels to restore blood flow. For patients admitted to hospitals with PCI capacity or transferred primary PCI <2 hours, primary PCI should be performed as soon as possible. Thrombolysis can still be considered for patients first diagnosed in non-PCI institutions with transport time >2 hours, but its efficacy remains to be discussed and further verified.

## 1. Introduction

Electrocardiogram (ECG) examination is the first choice for patients with chest pain in the emergency department. The vast majority of patients with acute coronary syndrome can be quickly identified by ECG, but there are still 30% of patients with acute coronary artery lesions that cannot be recognized by ECG in time, resulting in delayed treatment. de Winter syndrome was first proposed in 2008, accounting for about 2% of patients with anterior descending occlusion lesions, and is easily ignored by clinicians.^[1,2]^ It has not been recommended in the latest European and American guidelines^[3,4]^ due to their specific manifestations. Chinese Guidelines for the Diagnosis and Treatment of Acute ST-segment Elevation Myocardial Infarction (2019) recommend de Winter syndrome patients to be treated as a special type of ST-segment elevation myocardial infarction.^[5]^ However, there is no guideline to recommend whether intravenous thrombolytic therapy should be preferred for de Winter patients with chest pain first diagnosed in non-PCI (percutaneous coronary intervention) institutions. Herein, we report a case of de Winter syndrome with poor thrombolytic effect to explore the optimal emergency management strategy for this patient.

## 2. Case presentation

**Figure 1.**
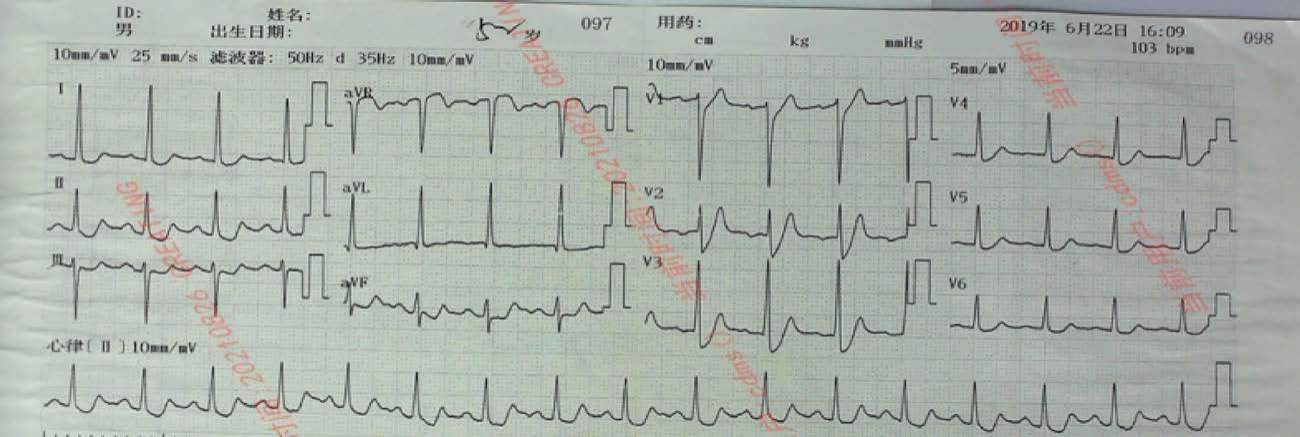
Electrocardiogram of the patient at the first visit: upward ST-segment of lead V2 to V5 in the precordial leads with j-points depressed 2 to 4 mV.

**Figure 2.**
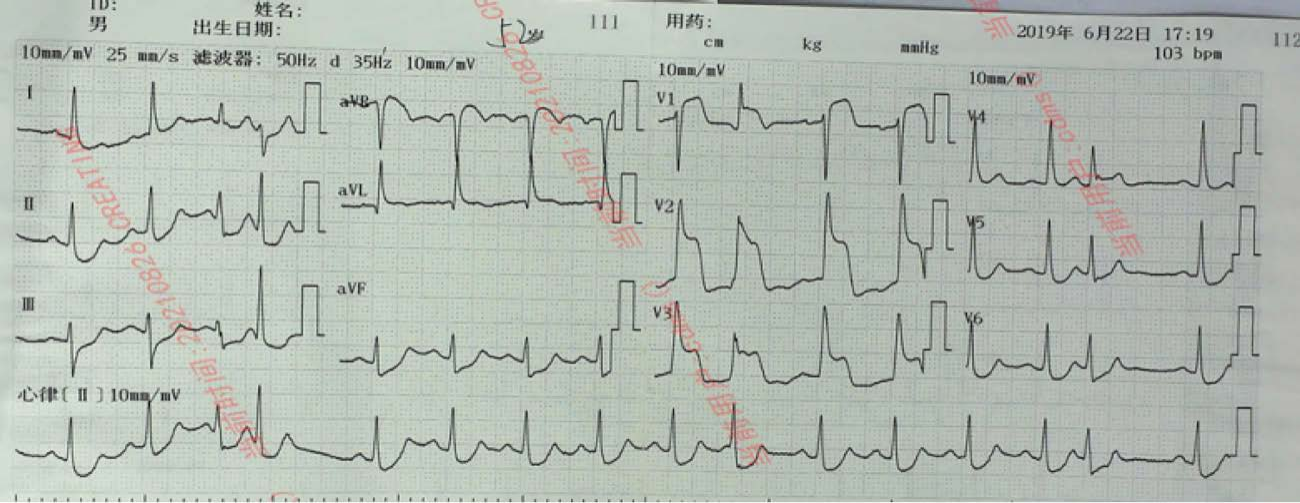
Recurrence of chest pain after the first thrombolytic therapy, reexamination of electrocardiogram: ST-segment elevation 2 to 10 mV in the precordial leads.

**Figure 3.**
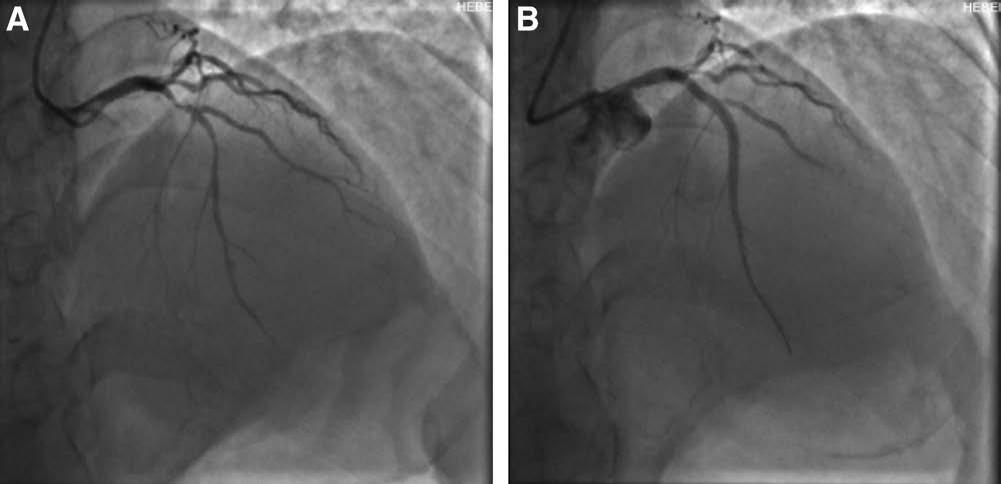
Coronary angiography indicating severe stenosis of the proximal anterior descending branch (A), while blood flow was significantly recovered after stent implantation (B).

**Figure 4.**
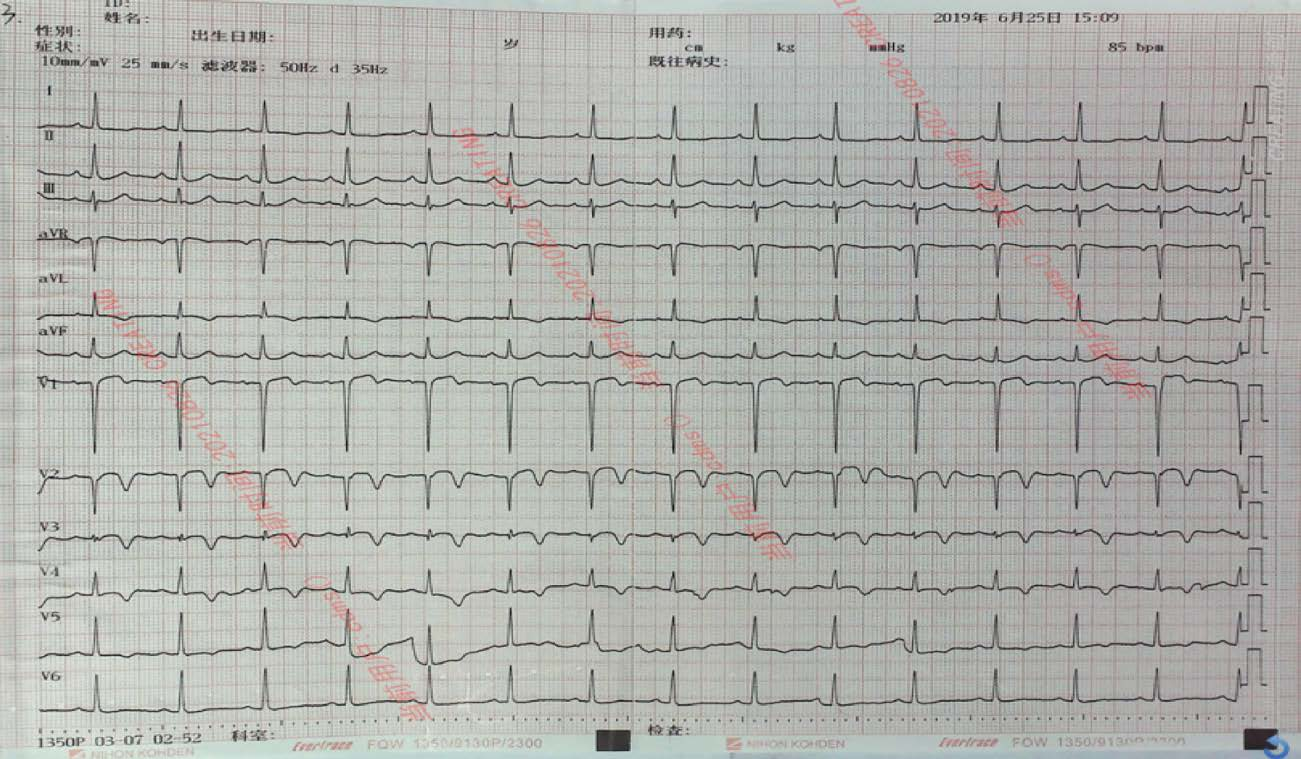
In-hospital electrocardiogram of the patient suggested dynamic evolution of the anterior wall, with the formation of Q-wave in the leads V1 to V2 and inversion of T-wave in the leads V1 to V5.

A 51-year-old male patient was admitted to the emergency department of our hospital for more than 5 hours due to intermittent chest tightness for 1 week. Previous healthy, no smoking or drinking history. Five hours ago, the patient developed chest tightness without clear cause, accompanied by chest pain, burning sensation in the precardiac area and dyspnea. The symptoms continued without relief, and the patient visited a local hospital without PCI capability. An ECG at local hospital showed a de Winter pattern (Fig. 1). The patient received subcutaneous anticoagulant therapy (nadroparin 3000U) and dual antiplatelet (aspirin 300 mg and clopidogrel 300mg). The patient subsequently received intravenous thrombolytic therapy with reteplase (30 minutes interval, 10 wu, a total of 2 times). After the second administration, the patient had chest pain again, and the ECG (Fig. 2) showed ST segment elevation of the anterior wall lead. The patient was transferred to our hospital for rescue PCI. The angiography results suggested diffuse stenosis in the proximal to middle of the left anterior descending (LAD) artery, with the most severe stenosis at the proximal LAD artery, about 95% stenosis, and blood flow thrombolysis in myocardial infarction level 2 (Fig. 3A). Two stents were implanted in the proximal to middle part of the LAD, and post-procedure angiography indicated significant improvement in LAD blood flow (Fig. 3B). Post-procedure ECG showed T wave inversion and Q wave formation in the precordial leads. Color Doppler echocardiography showed decreased anterior wall movement of left ventricle and impaired systolic function of left ventricle. In-hospital ECG showed typical dynamic evolution after myocardial infarction (Fig. 4).

## 3. Discussion

Typical clinical symptoms and ST-segment elevation on ECG can accurately indicate complete coronary artery occlusion and the resulting transmural myocardial ischemia, which urgently requires primary coronary intervention. Currently, the door-to-balloon time is required to be within 90 minutes. Thrombolysis is preferred when the first diagnosis is made at a non-PCI facility and the transit time to PCI exceeds 120 minutes.^[4]^ Despite the low incidence, patients with de Winter syndrome have no better short-term prognosis than patients with anterior wall myocardial infarction.^[6]^ Therefore, de Winter syndrome should be managed as a high-risk ST-segment elevation myocardial infarction to improve its prognosis.

de Winter pattern has high specificity in predicting acute occlusion of proximal LAD. An analysis of 35 patients with de Winter pattern had a positive predictive value of 100% (95% confidence interval: 69.2–100.0%), and all patients had angiographic evidence of LAD obstruction.^[2]^

This phenomenon was observed in 6 of 330 patients diagnosed with non-ST-segment elevation myocardial infarction, all of whom had criminal lesions of LAD, with a positive predictive value of 100% (95% confidence interval: 51.7–100%).^[7]^ de Winter syndrome is highly predictive of coronary artery occlusion, which requires emergency opening of the occluded coronary artery.

de Winter syndrome is more likely to be associated with smoking,^[8–11]^ hypercholesterolemia,^[8]^ and uncontrolled hypertension,^[9,11]^ all of which have been shown in retrospective studies of de Winter syndrome. Cases of de Winter syndrome failed to recognized and effective reperfusion in emergency rooms were also reported in the real world.^[12]^ Early identification of de Winter syndrome, early angiography showing complete or incomplete occlusion of the LAD, and post-procedure recovery of the phenomenon suggest a better prognosis.

The preferred reperfusion strategy for patients with de Winter pattern is controversial. The majority of de Winter syndrome patients reported so far were first presented at a hospital with primary PCI capability and received primary PCI, achieving maximum ischemic improvement. In Xu’s study,^[13]^ 2 patients with de Winter pattern received intravenous thrombolytic therapy and showed poor effect, one of which developed coronary artery occlusion again after successful fibrinolysis. The authors observed 20 de Winter syndrome patients, and 3 of them were transferred to remedial PCI due to thrombolytic failure,^[6]^ which was also consistent with Xu’s study. Thrombolysis is an option for patients who are first admitted to an non-PCI hospital and transport time is >2 hours. In the case of Pranata et al,^[9]^ a 65-year-old Indonesian male with de Winter pattern was admitted to a non-PCI hospital. After intravenous thrombolytic therapy with streptokinase, the symptoms were significantly improved and ST-segment on the ECG dropped to the limit level. Shergill et al reported a 34-year-old Indian male smoker who presented with de Winter pattern on his ECG due to angina pectoris, whose symptoms were relieved ECG returned to baseline with streptokinase thrombolysis.^[14]^ Both patients were treated with thrombolytic therapy because coronary angiography could not be performed, and their symptoms improved and the ST-segment of the ECG retreated. Therefore, further evidence is needed to confirm whether thrombolytic therapy is recommended in de Winter syndrome.

## 4. Conclusion

de Winter syndrome, although low in incidence (about 2%), is a strong indication of severe coronary artery disease, requiring rapid identification and opening of coronary vessels to restore blood flow. For patients admitted to hospitals with PCI capacity or transferred primary PCI <2 hours, primary PCI should be performed as soon as possible. Thrombolysis can still be considered for patients first diagnosed in non-PCI institutions with transport time >2 hours, but its efficacy remains to be discussed and further verified.

## Author contributions

**Data curation:** Hao Xiao.

**Formal analysis:** Hao Xiao.

**Funding acquisition:** Li Shuren.

**Investigation:** Zhang Feifei.

**Software:** Hao Xiao, Zhao Mei.

**Supervision:** Zhao Mei, Liu Huiliang.

**Writing – original draft:** Hao Xiao.

**Writing – review & editing:** Hao Xiao.
